# Developing New Methods for Person-Centred Approaches to Adjudicate Context–Mechanism–Outcome Configurations in Realist Evaluation

**DOI:** 10.3390/ijerph19042370

**Published:** 2022-02-18

**Authors:** Seán Paul Teeling, Jan Dewing, Deborah Baldie

**Affiliations:** 1 UCD Centre for Interdisciplinary Research, Education and Innovation in Health Systems, School of Nursing, Midwifery & Health Systems, University College Dublin, D04 V1W8 Dublin, Ireland; 2 Mater Misericordiae University Hospital, Eccles Street, D07 AX57 Dublin, Ireland; 3 Centre for Person-Centred Practice Research Division of Nursing, School of Health Sciences, Queen Margaret University, Queen Margaret University Drive, Musselburgh, East Lothian EH21 6UU, UK; jdewing@qmu.ac.uk (J.D.); dbaldie@qmu.ac.uk (D.B.); 4 NHS Grampian, Aberdeen AB25 2ZN, UK

**Keywords:** realist evaluation, creative methods, person centred, person-centred cultures, data collection, Lean Six Sigma

## Abstract

Realist evaluation provides a general method of evaluating the application of interventions including policy, legislation, projects, and new processes in social settings such as law enforcement, healthcare and education. Realist evaluation focuses on what about interventions works, for whom, and in what circumstances, and there is a growing body of work using realist evaluation to analyse interventions in healthcare organizations, including those using Lean Six Sigma improvement methodologies. Whilst realist evaluation facilitates the analysis of interventions using both qualitative and quantitative research, there is little guidance given on methods of data collection and analysis. The purpose of this study is to address this lack of guidance through detailing the use of innovative person-centred methods of data collection and analysis in a realist evaluation that enabled us to understand the contribution of Lean Six Sigma to person-centred care and cultures. This use of person-centred principles in the adjudication of identified program theories has informed novel methods of collecting and analysing data in realist evaluation that facilitate a person-centred approach to working with research participants and a way of making the implicit explicit when adjudicating program theory.

## 1. Introduction

### 1.1. Background

As part of our research into the influence of Lean Six Sigma (LSS) on person-centredness, in a recent realist evaluation [1], we addressed whether, to what extent, and in what ways the process improvement methodologies of Lean and Six Sigma in healthcare contributed to person-centred care and cultures. Through realist evaluation, we were able to understand how healthcare staff who were LSS practitioners and had undertaken a university LSS education and training program understood and experienced, in their specific contexts of practice, the contribution of the application of LSS learning and practice to person-centred care and cultures. The realist evaluation was undertaken at a university teaching hospital in Dublin Ireland, a major centre for medical, nursing, and allied health professional training and a teaching partner to University College Dublin (UCD) since its foundation. Between 2014 and 2020, a LSS staff education and training program, a joint undertaking between UCD and the study site [2], has delivered over two hundred process and quality improvement projects to improve patient outcomes and patient and staff experiences of care, in over fifty healthcare institutions and Community Healthcare Organisations (CHOs) in Ireland. This paper does not repeat the findings of our study [1] but rather outlines how we addressed the problem of applying new methods of collecting and analysing data within realist evaluation that facilitated person-centred ways of working with research participants.

### 1.2. Person-Centredness

Person-centredness is a term that is now internationally recognised within health and social care. McCormack and McCance [3] describe person-centredness as:

An approach to practice established through the formation and fostering of therapeutic relationships between all care providers, service users and others significant to them in their lives. It is underpinned by values of respect for persons (personhood), individual right to self-determination, mutual respect, and understanding. It is enabled by cultures of empowerment that foster continuous approaches to practice development (page 3).

Person-centredness refers to embedded practices within a specific type of culture that enables and facilitates the delivery of person-centred care [4]. McCormack and McCance [3] claim that health professionals need to have their own personhood acknowledged as equal to that of patients, in effect to experience person-centredness. Person-centredness can therefore be viewed as the ‘operationalisation of personhood’ [5]. Applied to patients, it can be considered ‘person-centred care’; and when it is applied to patients and others, it is termed person-centred practice. Achieving person-centred care is reliant on person-centred cultures that enable all persons involved in the care process to flourish within health systems [6]. A person-centred culture involves the adoption of person-centred practice inclusive of all stakeholders within the health system. Person-centredness emphasises the development of person-centred cultures through the use of collaborative, inclusive, and participatory (CIP) principles [7,8]. Person-centred cultures are themselves necessary for the delivery of person-centred care [3]. Person-centred care has an explicit focus on ensuring the client or patient is at the centre of care delivery [6] and is concerned with every person involved in the patient’s care, not just the patient [3]. In practice, person-centredness is manifested in what Phelan and colleagues [9] term ‘compassionate care relationships’, wherein the healthcare provider focuses on what the customer/service user needs. Internationally, whilst there is a body of research on LSS, there is little research on its specific influence on person-centredness.

### 1.3. Lean Six Sigma

LSS has been used in healthcare since 2001 in the United Kingdom (UK) and since 2002 in the United States of America (USA) with Lean, Six Sigma, and LSS now considered to be some of the most popular process improvement methodologies in healthcare internationally [10,11,12]. Similarly, since the millennium, political and policy stakeholders have widely advocated that person-centred care should be at the heart of the health service [13,14,15,16]. LSS merges both Lean and Six Sigma process improvement methodologies [17] and works well in traditional process-driven settings such as production by removing non-value-added activity, reducing variation, standardising procedures, and subsequently reducing costs [17]. It has also been evidenced to be highly effective in healthcare [18,19,20,21,22,23], affecting processes of care, quality of care, finances, and patient and staff satisfaction [24], and has been shown to be synergistic with elements of person-centred approaches to improvement in theory [1,25] and practice [26,27]. LSS gives structure to process improvement through the defined steps of Define, Measure, Analyse, Improve and Control (DMAIC), facilitating a structured approach that allows for evaluation and re-evaluation of process improvement outcomes [1,25]. Evaluation and re-evaluation is inherent in the Control Phase, which mirrors the Model for Improvement Plan Do Check Act (PDCA) Deming wheel or Shewhart’s cycle, a quality improvement methodology that allows for both single-loop learning, where people, organisations or groups modify their actions according to the difference between expected and reached outcomes; and double-loop learning, where they modify their actions but additionally correct or change the underlying causes behind identified problems [25]. Both Lean and Six Sigma methodologies have a strong focus on the customer, the employee, management support and teamwork [1,11,24,25]. A key strength of LSS is that it seeks to find the ‘root cause’ of problems in a process, which means that it utilises real-time observational data collection, the process of which is referred to as ‘Gemba’ in Lean terminology [1,25]. This form of non-judgmental observational study is not unique to LSS, with workplace observations being utilised in practice development [3,25] to measure and evaluate ‘where we are now’ and has been illustrated to be a key synergy when using both LSS and person-centred approaches to improvement [1,25]. 

### 1.4. Study Participants

A purposive sample of qualified LSS practitioners (*n* = 20) at the study site identified different contexts (C) which, with key mechanisms (M), were considered to trigger/ prevent a range of outcomes (O) where the intervention of the LSS education and training program was introduced. Consistent with realist evaluation methodology, participants helped construct, confirm, refute, or refine the program theory [28] and identified what, in their experience, facilitated or hindered the effectiveness of the intervention to deliver anticipated outcomes [29]. Additionally, in keeping with realist evaluation, our research team featured a core expert group [30]. This group included three members who both practiced in and lectured on person-centred practice research, person-centred cultures and care [1]. It was therefore philosophically and methodologically important for us that a key feature of our data collection process within the realist evaluation was the authentic commitment by us to the person-centred principles of collaborative, inclusive, and participatory (CIP) ways of working [7,8] to underpin the approach to data collection to gather participants’ views and experiences as LSS practitioners.

### 1.5. Realist Evaluation

Realist evaluation is a theory-based evaluation designed to test and refine a theory that has informed the development of multiple and varied programs or interventions. Realist evaluation aims to understand ‘what works, for whom, in what circumstances and why’ [28]. This means rather than solely providing judgments on a program’s success or failure, there is an appreciation that the underlying causative factors must also be investigated and understood. Conversely, traditional evaluation approaches attempt to estimate program effectiveness through the assessment of program outcomes [31,32]. This approach to evaluation, sometimes called ‘black box evaluation’ [33] focuses on outcomes without seeking to understand how the outcome was achieved. Pawson [34] sees realist evaluation as a form of theory-based evaluation specifically developed to strengthen the explanatory power of evaluation studies. Theory-based evaluations are, according to Hansen [35], unlike other result and process evaluation models of evaluation, as they focus not only on outcome measurements but also on the mechanisms and contexts that support, or hinder, the realisation of those outcomes. From a realist evaluation viewpoint, a CMO configuration (CMOc) can be seen as a hypothesis that a program outcome (O) emerges because of the action of underlying mechanisms (M), which are activated only in particular contexts (C). Pawson and Tilley [28] see realist inquiry as enabling researchers to investigate the world from a realist perspective with a focus on the development and refinement of CMOcs. However, Pawson [36] states that the activation of the mechanism is dependent on variables such as individual characteristics, circumstances, and situations: the context (C), and variation in mechanisms, leads to variation in outcomes (O). This approach to evaluating social programs enables the theories within a program to be made explicit, by developing clear hypotheses about how, and for whom, programs might work, and in what context. Realist evaluations, in general, begin with an initial program theory (hypothesis) and end with a more developed theory [37]; therefore, the first step in conducting realist evaluation is to develop the program theory to explain how a proposed intervention is expected to work in the eyes of the program designers and implementers [28]. For this study, we drew on the expertise of an expert reference group to develop an initial, high-level, program theory: LSS can have a positive influence on person-centred care and person-centred cultures if delivered through the intervention of a LSS staff education and training program [1]. We sought to understand the influence of LSS on staff through exploring staffs’ lived experiences of engaging with the program and using the data that generated to collectively with research participants, adjudicate the theory and evidence-informed CMOc.

There are many advantages to the use of realist evaluation in health systems research. Firstly, against the ‘black box’ theory [33], realist evaluation provides a basis for the analysis of the influence of context and mechanism on outcomes [29,38]. Whilst healthcare is known for its complexity [39] rather than seeing realist evaluation as lacking the capacity to tackle complexity, it has been and continues to be used in complex healthcare situations and with complex interventions [38,40]. Realist approaches have been widely used to understand complex issues such as patient and public involvement [41], improving care for frail older patients [42], and the effectiveness of Quality Care Process Metrics in Nursing and Midwifery services [43].

### 1.6. The Problem 

A key strength of realist evaluation as the methodological approach for our recent study [1] was its use of both qualitative and quantitative research [44,45,46]. Realist evaluation values mixed-methods approaches and suggests that data types should be selected for their potential contribution to the research. This mixed-methods approach allows for data collection that involves the collection, analysis, and interpretation of both quantitative and qualitative data in a single study [47], allowing for the study of complex interactions [48]. In carrying out the realist evaluation [1], we were informed by the Realist And Meta-narrative Evidence Syntheses: Evolving Standards (RAMESES II) reporting guidelines for realist evaluations [29]. However, we found that that there was and is little guidance given on the methodological processes to be used in actual data collection [49]. Salter and Kothari [50] similarly found that little guidance exists on any particular approach to analysis within realist evaluation. Gilmore and colleagues [49] suggest that, despite the increase in the number of healthcare-related realist evaluations, few publications provide details of the methodological processes used. The problem we therefore faced was in developing data collection methods for our study so that they would be both appropriate for use in realist evaluation whilst consistent with CIP principles [7,8]. We now detail how we addressed the problem of applying new methods of collecting and analysing data within realist evaluation that facilitated person-centred ways of working with research participants

## 2. Methods

### 2.1. Using Person-Centred Principles

Data collection that involved a direct participant interface included a series of facilitated workshops with study participants (*n* = 20) to gather participants’ views and experiences of using LSS within their practice areas as LSS practitioners. The sample size was purposive (*n* = 20), derived from hospital staff from a range of disciplines and functions, all active LSS practitioners and graduates of a University LSS education and training program, having graduated between 2014 and 2017, and currently working in the study site. This sample constituted 20% (*n* = 97) of the population of LSS graduate practitioners in the study site and was a feasible number of participants to work with. Graduates of the LSS education and training program were chosen in line with realist evaluation principles, with Pawson and Tilley [28] highlighting that they probably would have experienced both the successes and failures of the program intervention (the LSS education and training program) and would be best placed to advise on outcomes. The workshops were followed by individual semi-structured realist interviews [51] and a second series of facilitated workshops with study participants to arrive at a final adjudication of the draft program theories resulting from analysis of both the first series of workshops and the interviews. The methods used within the workshops and interviews were underpinned by person-centred principles CIP ways of working [7,8]. Person-centred approaches are both creative and critical [52], with creative methods offering a different way to explore the research question with participants as they enable deeper thought on the question being discussed [53,54,55]. Kara [55] claims that although associated with arts-based methods, there is scope for the use of creativity within traditional research methods such as oral interviews and focus groups, both of which we have found can be used in realist evaluation. Creative methods additionally create time and space for research participants to reflect on complex issues and allow time for reflection giving each research participant time to think [56]. However, we were aware that although creative methods appealed to us as person-centred researchers and practitioners, it was essential that the creative methods chosen addressed the research question and were consistent with the study methodology [57]. These creative methods facilitated participant feedback and adjudication of the program theory, adjudication being the interrogation of underlying causal processes [58] and facilitated thematic analysis. The ultimate purpose of data analysis through adjudication is to identify whether participants inspire/validate/falsify/ modify [36] the program theory. The creative methods we used within this study are defined in Table 1.

We now proceed to discuss each of the utilised creative methods individually and in more detail, beginning with our use of artifacts.

### 2.2. The Use of Artifacts

**Figure 1:**
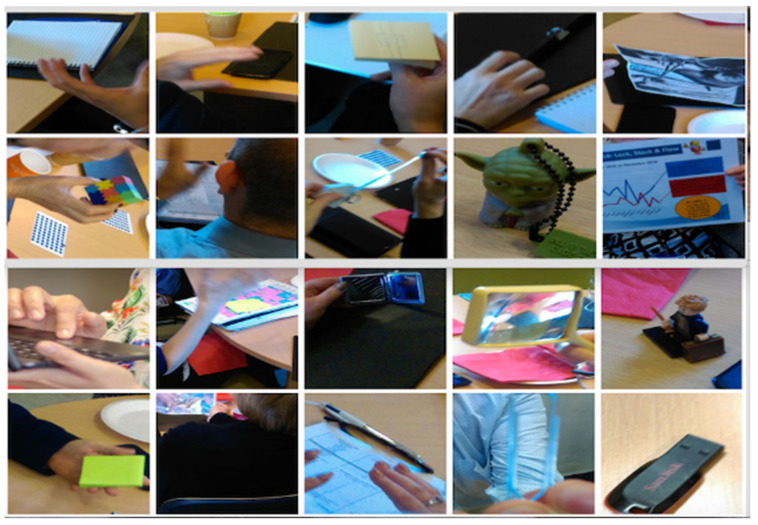
An example of participant artifacts (source authors).

The interview is a common method of data collection in qualitative research [59]. The use of semi-structured interviews within a realist structure [28] facilitated an interview format that allowed pre-determined topics to be covered; additionally, it also afforded the flexibility to discuss individual participant’s experiences in more detail. However, traditional semi-structured interviews do not always enable participants to answer questions in deep and meaningful ways. We, therefore, chose to augment our interviews with the use of artifacts. Before meeting research participants, we asked each of them to bring an artifact to the first workshop (Figure 1), which signified their current thoughts on their role as a LSS practitioner within their practice area. Following the first series of workshops, follow-up individual interviews were then conducted in the presence of the physical artifact/creation that the research participant had brought/created, with the artifacts seen to embody the knowledge, skills, and attitudes that the participant held. 

Artifacts are useful in qualitative data collection as they can assist in eliciting information that may not have been uncovered with traditional question and answer methods [60]. Artifact use can include (but is not limited to) pictures [61,62], poetry [63], painting and collage [64]. Within the course of the realist interview, research participants were invited to discuss, explain and articulate their individual thinking about their experiences as LSS practitioners and to use their experience to adjudicate and refine the CMOc. This is a collaborative approach to theory refinement in which the interview is guided by the theories the researcher is aiming to refine [28,51]. Within interviews, we found that the artifacts provided framing for the research participants to describe their experiences as LSS practitioners, and provided the interviewer with useful visual cues to support detailed follow-up questions to peel away layers of understanding and enable adjudication of program theories and development of CMOc. The artifacts were not only useful for and enhanced the interviews [65] but proved useful for initial introductions in workshops and enabled each participant to speak about their own experience and use of LSS within their practice areas. Within our data collection, we also made use of both display boards and participant workbooks, which we now discuss.

### 2.3. The Use of Display Boards and Workbooks

Workshops are an effective method of refining theory using realist evaluation [66] and we found that our workshops enabled what Pawson and Tilley [28] term pattern identification with participants in a ‘theory-testing role’. Rushmeer and colleagues [66], in evaluating a ‘knowledge to action’ program, noted that workshops can enable a ‘two-way process where knowledge, evidence, opinions and experiences of ‘what works’ are shared and discussed by stakeholders’ (p. 553). The workshops enabled this two-way process for us as researchers, enabling the CMOc to be adjudicated by the workshop participants and the program theory to be refined.

**Figure 2:**
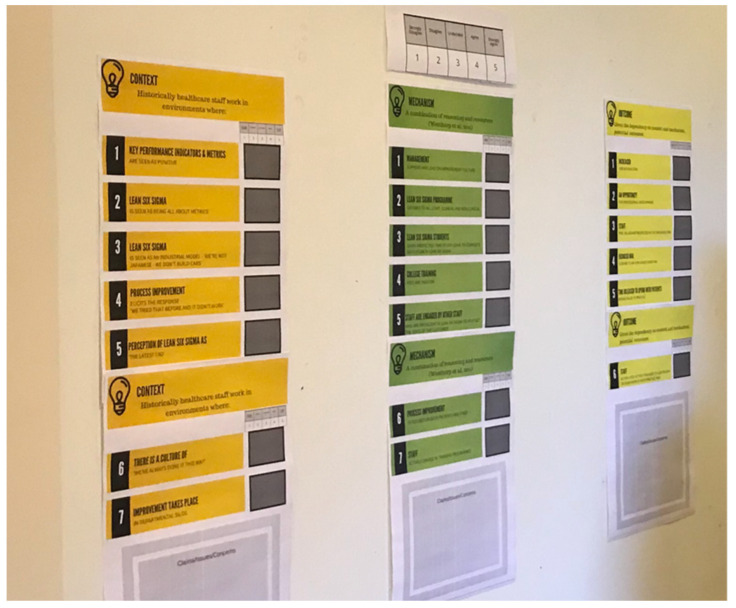
An example of display boards for workshops (source authors).

Effective facilitation of workshops can enable a range of participant outputs, including frank exchange and common understanding of different views on the issues being discussed and the effective communication of information from several sources [67]. Facilitators can therefore use a variety of strategies to support the workshop process while remaining neutral to the content of group discussions [68]. To enhance our facilitation of the workshops, we, therefore, introduced the use of colour-coded wall-mounted display boards (Figure 2) to our workshops, each containing the specific CMOc for discussion at the particular workshop. The Contexts, Mechanisms, and Outcomes derived from the literature [1] were shared with the participants using these display boards and were key to the review of the CMOc by participants throughout the workshops. The display boards were wipeable and allowed participants to write on them, being consistent with realist evaluation methodology by effectively enabling them to confirm, refute or refine the program theory [28] and identify what, in their experience, facilitated or hindered the effectiveness of the intervention to deliver anticipated outcomes.

**Figure 3:**
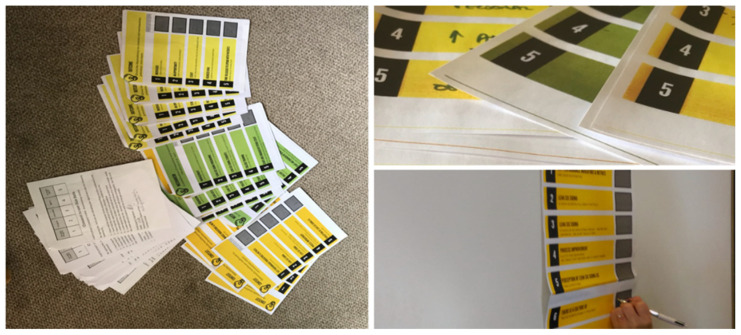
An example of our workbooks (source authors).

Researchers have found success with using workbooks for various situations, with users finding an emphasis on the graphical format and visual thinking a pleasant change from the typically written format [69]. Workbooks can be used to elucidate complicated concepts with more understandable easy reference notes and explanations. To supplement the display boards, we, therefore, designed individual participant workbooks each containing a section for Contexts, Mechanisms, and Outcomes (Figure 3). 

The workbooks were colour-coded to match the display boards for ease of identification of specific Contexts, Mechanisms, and Outcomes. The workbooks contained simple explanatory notes of each of the CMOc for discussion and review, and blank sections for personal notes. Participants were encouraged to take time to write their own thoughts on what Contexts, Mechanisms, and Outcomes were relevant to them in their daily practice as LSS practitioners, building on their personal artifacts. As facilitators, we remained on hand to offer clarification as required. This was critical to working in person-centred and, in essence, democratic ways as it offered each participant to consider their own experience and then use these to work with others collaboratively thus avoiding their views being initially formed or influenced by views of others as often can happen in focus groups for example. We now discuss our use of word clouds as a method for data collection.

### 2.4. The Use of Word Clouds

Word clouds are a useful tool for the analysis of textual data and enable provide meaningful interpretations through text size, dimension, and colour [70]. A word, concept, or term mentioned more frequently will be visualised in a larger font or text size in the finished word cloud and those mentioned less frequently will be included in a smaller font or will not be included at all. This visual representation or graphic portrays patterns of keywords and phrases included in the text, allowing research participants to identify relationships and meaning [70].

**Figure 4:**
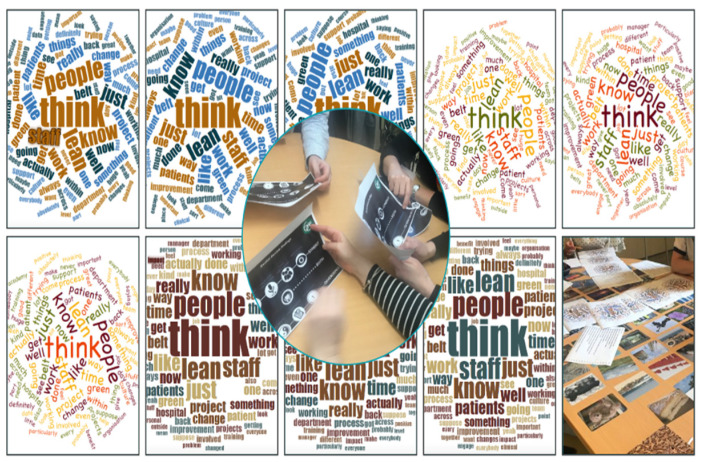
Example of CMOc word clouds from this study (source authors).

Word clouds are popular, fun ways to display text data in graphical form, enabling common themes to be identified and useful as a starting point or screening tool for large amounts of text data [71]. For our second series of workshops, each research participant was given an individual information pack that contained a map of all of the iterations of the CMOc to date and for accessibility a visual word cloud for each iteration (Figure 4) to facilitate reflection. Time was given for the individuals to read through the packs and reflect before discussing within small groups. The word clouds provided a visual reference to keywords and themes across all developments in the adjudication to date, from an initial realist review [1] through the first series of workshops to the individual interviews. 

The research team was on hand throughout to answer any clarifying questions concerning the materials or intent of the workshop and to assist as required. However, we did not interfere with the groups and left them to develop their own interpretation of the information packs and accompanying word clouds, offering input only if requested. We now outline our use of creative constructs.

### 2.5. The Use of Creative Constructs

**Figure 5:**
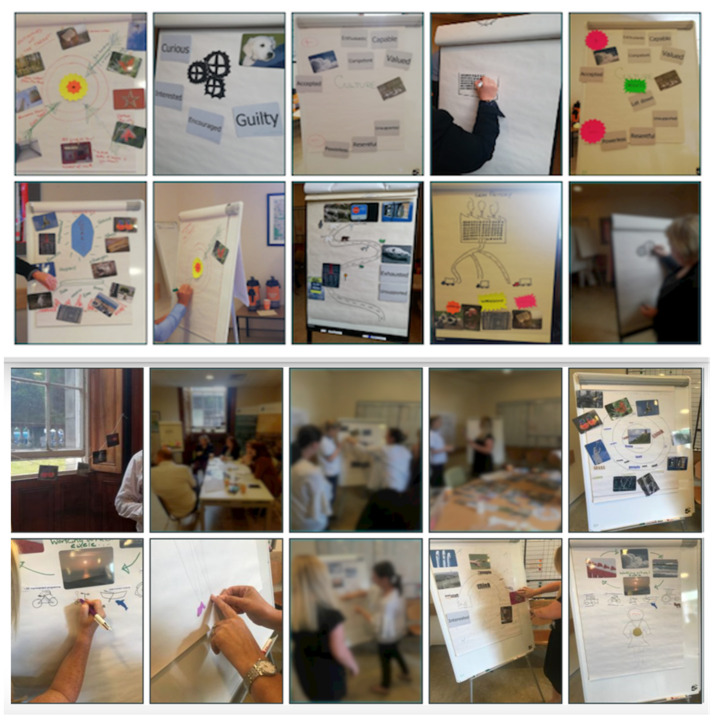
Creative constructs (source authors).

The person-centred CIP principles [7,8] continually underpinned the approach to the workshops to gather participants’ views and experiences as LSS practitioners. A range of creative approaches were used to achieve these collaborative and inclusive ways of working including the use of pictures and creative constructs [72] and other means such as the use of painting and collage [64,73]. These approaches facilitated participant feedback and adjudication of the program theory, adjudication being the interrogation of underlying causal processes [58] and further facilitated thematic analysis. We aimed to create a safe space in which to work together in interdisciplinary teams and to design a creative construct to represent teams’ further understanding and development of the CMOc. To facilitate this creativity boxes of creative materials, packs of Evoke© cards and flipcharts were provided. At the end of each workshop, the participating teams produced a visual construct for Contexts, Mechanisms, and Outcomes, based on their individual and team analysis of the CMO iterations to date (Figure 5). 

Participants reflected on this individually discussed it in teams and then presented their findings by mapping previously identified Synergies and Divergence between LSS and Person-centredness [1,25] to their final constructs using sticky notes. Throughout our workshops, we also made use of Evoke© cards.

### 2.6. The Use of Evoke© Cards

**Figure 6:**
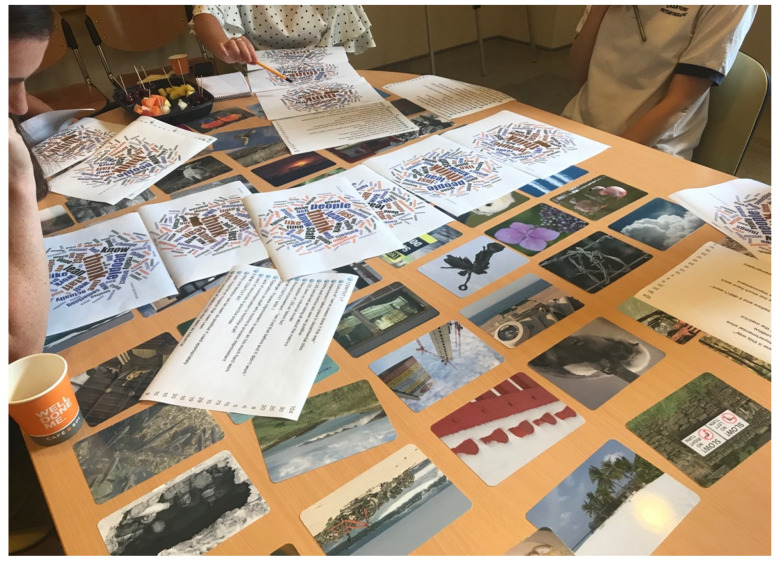
Use of Evoke© cards (source authors).

At the end of each workshop, a small ‘debriefing’ exercise was carried out using Evoke© cards (Figure 6). Evoke© is a deck of 72 cards containing an image on one side and wording on the other, that are intended to help to evoke a range of memories, reflections, or ideas [74]. They are intended as a creative method of exploring feelings and gaining insights into situations [75]. 

The use of picture cards such as Evoke© is a valuable addition to current methodologies for eliciting feedback [76]. Each participant chose a card to feedback on how they felt after the workshop. This was consistent with the duty of care held by the researcher to be mindful of participants’ feelings. We now detail our use of continuous reflection within this study.

### 2.7. Reflexivity

Throughout the research, we remained cognizant that our PI was both a researcher and a lecturer on the LSS education and training program (the intervention), a research insider, and that remained a concern for us and again was a continuous topic of discussion as a team. The use of reflexivity was important for this study as it related to methodology, person-centredness, and organisational change:

- Methodologically, the use of reflexivity is congruent with realist evaluation, utilising multiple data sources and methods pragmatically and reflexively to build a picture of the case, which calls for making sense of various data sets to develop coherent and plausible accounts of the phenomena under investigation [77,78].
- From a person-centred perspective, [79] indicates that reflexivity is a skill that person-centred leaders need to nurture.
- Concerning organisational change and development (such as the intervention of the LSS education and training program), reflective practice is highlighted as being a central part of the change process [80].

Throughout all cyclical stages of the research process, we reflected continuously on our interactions with participants and the interpersonal relationships involved, and maintained an awareness of the question of power and having a relationship with all participants based on trust, respect, and reciprocity [81]. Reflexivity was managed at each stage of this study and made use of the following methods:

- Reflection after each stage of the process was facilitated by Rolfe et al.’s [82] ‘What, So What, Now What’ model (Table 2), a reflective tool. The tool highlights areas for learning and development. It was particularly useful for us to reflect on each workshop so that the learning and program theory insights could feed forward to subsequent data collection.
- Consideration and reflection on our own listening skills, facilitated by Dewing et al.’s. (2014) process evaluation record, a simple tool to reflect on your individual listening skills, which was particularly useful at the individual interview stage.
- Use of personal research journals capturing our reflections on each stage of the process.
- Reflection and feedback with the community of practice for doctoral students (SICOP) at Queen Margaret University Edinburgh, following the interviews and before the final series of workshops.

## 3. Results

### 3.1. Successful Adjudication of Program Theory

The ultimate purpose of data analysis through adjudication is to identify whether participants ‘inspire/validate/falsify/ modify’ [36] the program theory. Through our use of creative methods, we were able to successfully develop three focused CMOc relating to staff [1] and participants were enabled to align these to previously identified synergies and divergences [25] between participants’ LSS practice and person-centred cultures. This enabled us to understand the contribution of LSS to person-centred care and cultures that contribute to the evidence base on the study of quality improvement beyond intervention effectiveness alone.

### 3.2. Successful Use of Creative Methods

The use of creative methods enabled us to ensure that data collection and analysis were underpinned by person-centred principles that are an effective and acceptable way of capturing the experiences of participants [83] and of facilitating authentic collaboration [84].

The person-centred approach and creative methods used in our study [1] allowed a valuable reflection space for the participants, all busy practitioners. The benefits afforded by creative methods included having the potential to develop a rapport with participants, providing them time and space to reflect more deeply on issues and to enable their thinking to be made visible [85] and offered a rationale for the use of person-centred approaches (Table 1) within the traditional research methods aligned to the critical realist paradigm.

### 3.3. Positive Reception of Methods from Research Participants

Finding what is working and not working about group work from different perspectives is a key part of a person-centred development work. Following our final workshop, we conducted a ‘what is working/not working’ [86] exercise with research participants (*n*= 20). This sorts what is working and what is not working from different participant perspectives to identify future ways of working. As a really simple way of analysing what happened in the workshops from the participant’s perspective, it can help to identify how the participants experienced the creative methods used. 100% of participants said that they had enjoyed the research process. More specific positive ‘what worked’ feedback from research participant’s included

- ‘Particularly liked the way we were able to be creative’.
- ‘What I thought would be a boring process was actually quite fun, but serious at the same time’.
- ‘Why isn’t more research conducted like this?’
- ‘The use of the constructs really got the conversation going and enabled robust discussion among the group’.
- ‘I expected to be enervated but actually, I was energized’.

What ‘didn’t work’ feedback from participants included

‘Time was an issue. I would have liked more time in the workshops to work on the creative piece and to expand on this a bit more’.

‘Some of us were more comfortable with the creative elements than others, but everybody enjoyed the experience’.

This feedback reinforced the value participants placed on these methods for enabling them to reflect on the issues being researched and to enable program theory adjudication. It also served as a reminder to us as researchers of allowing sufficient time for exploration of themes and further reflection and we have fed this forward to subsequent work we have undertaken with clinical teams [18,19,20,21].

## 4. Discussion

We undertook a realist evaluation to understand whether, to what extent, and in what ways LSS in healthcare contributes to person-centred care and cultures [1]. Because this study was investigating person-centredness, person-centred values and principles guided the design and conduct of the realist evaluation. However, both realist review and evaluation were used to evaluate the delivery of person-centred care in practice settings [73,87,88,89,90]. Therefore, our study was original in its combined use of person-centred and realist evaluation research methods to inform its design and implementation.

Our work has illustrated that it is possible to undertake research that has person-centredness at its philosophical and theoretical core and has foundations in multiple and mixed research methodologies to yield rich data sets that capture or pursue human experience [91]. As realist evaluation allows for multiple and mixed methods in research, it is possible to build person-centred processes into the research methods. The use of person-centred principles to guide data collection provided time for participants to reflect on the presented CMOc and to consider whether and to what extent their own ways of working and their experiences as LSS practitioners were reflected in and by it. The use of the principles also created a safe space for participants to examine their LSS practice, seen as an important element in successfully engaging and working with others to develop effective workplace cultures [92]. The collaborative, inclusive, and participatory work [7,8] was facilitated by the shared purpose of participants to adjudicate the program theory. Shared purpose results when a group of individuals align their belief systems or values with a common challenge, vision, or goal [93] (p. 5). Shared purpose has been shown to unify diverse groups in collaborative activity, enabling participants to work together creatively in the same direction (Manley et al., 2014). The practical significance of our methods for data collection has been shown to be relevant in realist evaluation; however, we believe our methods have relevance for other multiple and mixed-methods research methodologies that use a variety of data sources to provide rich, often complex, sets of data (or evidence) necessary to answer complex research questions [91].

Throughout this study, our engagement with research participants was supported by ongoing reflection and reflexivity, which are essential for responsible and ethical practice. We understood reflection was an ongoing constituent of practice rather than a technique [94] and saw reflexivity as a continuous and integral part of the research process [95]. Reflection is a vital component in the research cycle, in tandem with reflexivity and an account of how it is employed in any project, particularly in reference to service developments [96]. This researcher reflexivity enables engagement in critical self-reflection about any personal biases, preferences, and preconceptions with theorists, encouraging researchers to layout their prejudices in a reflexive process [97]. Hammersley and Atkinson [98] suggest that rather than trying to eliminate the effects of the researcher, reflexive researchers actually try to understand them. Denning and Verschelden [99] describe how interdisciplinary workshops and focus groups in healthcare research allow for collaborative conversations that enable the refinement of ideas. This format proved particularly useful in our realist evaluation as it spotlighted participants’ attitudes, language, and understanding of the CMOc within the framework of their own practice setting [100]. Kitzinger [100] suggests that the interactions between research participants and the researcher allow them to generate and answer their own and each other’s questions and to share common experiences, which further deepen understanding of the research topic. Our results illustrated that these workshops were energising and fun for both the participants and the research team.

We recognise that our work was not without limitations. Being creative with research methods is tied to constraints such as the budget for creative materials, time constraints as creativity takes time, and, courage to try new methods within a methodological approach. We were fortunate however that, although there was the possibility of poor acceptance of our methods by participants, they were overwhelmingly popular with our participants and enabled us to address our research question. We acknowledge that the creative methods we employed are but a small fraction of the creative tools available for use by researchers [55] but suggest we used those that best facilitated our particular context. Blamey and Mackenzie state that realist evaluation can be both time and resource intensive for the researcher [101] and given the lack of guidance on data collection and analysis in realist evaluation [49,50], much of this time can be attributed to developing data collection methods. We contend that this paper, with its guidance on person-centred methods for data collection, may save researchers some time in developing data collection methods, allowing for successful completion of the research within the time and resources available.

We have demonstrated that being true to an existing research methodology, in this case, realist evaluation, did not mean personhood was disregarded. Regardless of the research paradigm and philosophical framework, we have illustrated that data can be collected in person-centred ways and be focused on answering research questions grounded in person-centredness [91]. We identified that there was and is little guidance given on the methods for actual data collection or approaches to data analysis within realist evaluation [49,50]. This paper therefore offers valuable guidance for realist evaluators on how incorporating person-centredness results in a new way of adjudicating CMOcs and novel methods of working with research participants to collect and analyse data for realist evaluation. The use of collaborative, inclusive, participatory person-centred processes within our data collection was essential in both working with and gaining an understanding of the research participants’ deeply held values, beliefs, and experiences as they related to the research question. What was essential was that we aligned the philosophical underpinnings of the research and the deployment of the realist evaluation methodology and use of person-centred methods.

## 5. Conclusions

This study has shown that whilst adhering to the principles and rigor of realist evaluation, the use of person-centred principles with their inherent critical creativity [52] is appropriate for data collection and can be threaded through a realist evaluation approach [91]. This paper, therefore, contributes to theory and research in the areas of both person-centredness and realist evaluation. This in turn can influence the use of both in future practice. The use of person-centred principles to inform the adjudication of the CMOcs has led to novel methods of collecting and analysing data for realist evaluation that enact person-centredness in working with research participants. These methods can be adopted by realist evaluators who wish to work in a person-centred way whilst still adhering to the principles and rigor of realist evaluation.

## Figures and Tables

**Table 1:** Use of creative methods within our study.

Method	Used for	Used in
Use of artifacts	● Introducing self to others in the group and opportunity to make overt what matters to them as persons ● Facilitating in-person relationships with participants before later follow-up interviews ● Providing a framing for the research participants to describe their experiences as LSS practitioners	● Workshop series 1 ● Workshop series 2 ● Semi-structured interviews
Use of display boards creative workbooks	● Providing a means for research participants to note further, deeper knowing and suggestions not articulated verbally or arising from cognitive thinking alone ● Providing an adjunct for research participants to confirm, refine or refute program theory	● Workshop series 1 ● Workshop series 2
Use of word clouds	● Allowing research participants to identify relationships and meaning in the CMOc development to date	● Workshop series 2
Use of creative constructs	● Facilitated participant feedback and adjudication of the program theory ● Facilitated thematic analysis	● Workshop series 2
Use of Evoke© cards	● Providing a creative method of exploring feelings and gaining insights into experiences of engaging with the intervention	● Workshop series 1 ● Interviews ● Workshop series 2
Use of reflexivity by researchers and participants	● Making sense of various data sets to develop coherent and plausible accounts of the phenomena under investigation ● Maintained an awareness of the question of power and having a relationship with all participants	● Workshop series 1 ● Interviews ● Workshop series 2
Use of group process evaluation to identify what is working, what is not working	● Helped to identify how the participants experienced the combination of cognitive and creative methods used	● Post-workshop 2

**Table 2:** ‘What, So What, Now What’ questions.

Stage	Details
‘What’	Describe the situation; achievements, consequences, responses, feelings, and problems
‘So What?’	Discuss what has been learnt; learning about self, relationships, models, attitudes, cultures, actions, thoughts, understanding, and improvements
‘Now what?’	Identify what needs to be done in order to improve future outcomes and develop learning

## Data Availability

The data presented in this study are available in this paper.

## References

[1] Teeling S.P., Dewing J., Baldie D. (2021). A Realist Inquiry to Identify the Contribution of Lean Six Sigma to Person-Centred Care and Cultures. Int. J. Environ. Res. Public Health.

[2] McNamara M., Teeling S.P. (2019). Developing a University-Accredited Lean Six Sigma Curriculum to Overcome System Blindness. Int. J. Qual. Health Care.

[3] McCormack B., McCance T., Klopper H. (2017). Person-Centred Practice in Nursing and Health Care: Theory and Practice, 2nd ed.

[4] Hardiman M., Dewing J. (2019). Using Two Models of Workplace Facilitation to Create Conditions for Development of a Person-Centred Culture: A Participatory Action Research Study. J. Clin. Nurs..

[5] Anker-Hansen C., Skovdahl K., McCormack B., Tonnessen S. (2019). Collaboration between home care staff, leaders and care partners of older people with mental health problems: A focus on personhood. Scand. J. Caring Sci..

[6] McCormack B., Borg M., Cardiff S., Dewing J., Jacobs G., Janes N., Karlsson B., McCance T., Mekki T.E., Porock D. (2015). “Person-centredness—the ‘State’ of the Art. Int. Pract. Dev. J..

[7] Manley K., O’Keefe H., Jackson C., Pearce J., Smith S. (2014). A Shared Purpose Framework to Deliver Person-Centred, Safe and Effective Care: Organizational Transformation Using Practice Development Methodology. Int. Pract. Dev. J..

[8] Dewing J., McCormack B., Titchen A. (2015). Developing a Shared Vision for Person-Centred Care. Practice Development for Nursing, Health, and Social Care Teams.

[9] Phelan A., McCormack B., Dewing J., Brown D., Cardiff S., Cook N., Dickson C., Kmete S., Lorber M., Magowan R. (2020). Review of Developments in Person-Centred Healthcare. Int. Pract. Dev. J..

[10] Radnor Z., Osborne S.P. (2013). Lean: A Failed Theory for Public Services?. Public Manag. Rev..

[11] Williams S. Lean and Person-Centred Care: Are They at Odds?. http://www.pomsmeetings.org/ConfProceedings/051/FullPapers/Final%20Full%20length%20Papers/051-0066.pdf.

[12] Jorma T., Tiirinki H., Bloigu R., Turkki L. (2016). Lean Thinking in Finnish Healthcare. Leadersh. Health Serv..

[13] Bartz C.C. (2010). International Council of Nurses and Person-Centered Care. Int. J. Integr. Care.

[14] World Health Organization (2016). Framework on Integrated, People-Centred Health Services.

[15] (2019). International Council of Nursing Strategic Priorities-Person Centred Care. I.C.N..

[16] Nolte E. (2017). Implementing person centred approaches. BMJ.

[17] Black J. (2009). Transforming the Patient Care Environment with Lean Six Sigma and Realistic Evaluation. J. Healthc. Qual..

[18] Dempsey A., Robinson C., Moffatt N., Hennessy T., Bradshaw A. (2021). Lean Six Sigma Redesign of a Process for Healthcare Mandatory Education in Basic Life Support—A Pilot Study. Int. J. Environ. Res. Public. Health.

[19] Daly A., Wolfe N. (2021). Redesigning the Process for Scheduling Elective Orthopaedic Surgery: A Combined Lean Six Sigma and Person-Centred Approach. Int. J. Environ. Res. Public Health.

[20] O’Mahony L., McCarthy K. (2021). Using Lean Six Sigma to Redesign the Supply Chain to the Operating Room Department of a Private Hospital to Reduce Associated Costs and Release Nursing Time to Care. Int. J. Environ. Res. Public Health.

[21] Egan P., Pierce A., Flynn A. (2021). Releasing Operating Room Nursing Time to Care through the Reduction of Surgical Case Preparation Time: A Lean Six Sigma Pilot Study. Int. J. Environ. Res. Public Health.

[22] Creed M., McGuirk M., Buckley R., Kilduff M. (2019). Using Lean Six Sigma to Improve Controlled Drug Processes and Release Nursing Time. J. Nurs. Care Qual..

[23] Teeling S.P., Coetzee H., Phillips M., McKiernan M., Ní She É., Igoe A. (2019). Reducing Risk of Development or Exacerbation of Nutritional Deficits by Optimizing Patient Access to Mealtime Assistance. Int. J. Qual. Health Care.

[24] Deblois S., Lepanto L. (2016). Lean and Six Sigma in acute care: A systematic review of reviews. Int. J. Health Care Qual. Assur..

[25] Teeling S.P., Dewing J., Baldie D. (2020). A Discussion of the Synergy and Divergence between Lean Six Sigma and Person-Centred Improvement Sciences. Int. J. Res. Nurs..

[26] Connolly K., Teeling S.P., McNamara M. (2020). Live Well After Stroke. Int. Pract. Dev. J..

[27] Donegan D., Teeling S.P., McNamara M., McAweeney E., McGrory L., Mooney R. (2021). How Collaborative Working Reduced Older Persons’ Length of Stay in Acute Care and Increased Home Discharge: Calling Time on the ‘Dance of the Blind Reflex’. Int. Pract. Dev. J..

[28] Pawson R., Tilley N. (1997). Realistic Evaluation.

[29] Wong G., Westhorp G., Manzano A., Greenhalgh J., Jagosh J., Greenhalgh T. (2016). RAMESES II Reporting Standards for Realist Evaluations. BMC Med..

[30] Wong G., Westhorp G., Greenhalgh J., Manzano A., Jagosh J., Greenhalgh T. (2017). Quality and Reporting Standards, Resources, Training Materials and Information for Realist Evaluation: The RAMESES II Project. Health Serv. Deliv. Res..

[31] Connelly J.B., Duaso M.J., Butler G. (2007). A systematic review of controlled trials of interventions to prevent childhood obesity and overweight: A realistic synthesis of the evidence. Public Health.

[32] Hewitt G., Sims S., Harris R. (2012). The realist approach to evaluation research: An introduction. Int. J. Ther. Rehabil..

[33] Scriven M. (1994). The fine line between evaluation and explanation. Am. J. Eval..

[34] Pawson R. (2002). Evidence-Based Policy: The Promise of ‘Realist Synthesis’. Evaluation.

[35] Hansen H. (2005). Choosing evaluation models. Evaluation.

[36] Pawson R. (2006). Evidence-Based Policy A Realist Perspective.

[37] Birckmayer J.D., Weiss C.H. (2000). Theory-Based Evaluation in Practice. What Do We Learn?. Eval. Rev..

[38] Manzano-Santaella A. (2011). A Realistic Evaluation of Fines for Hospital Discharges: Incorporating the History of Programme Evaluations in the Analysis. Evaluation.

[39] Deming W.E. (2000). The New Economics for Industry, Government, Education.

[40] Pawson R., Wong G., Owen L. (2000). Myths, facts, and conditional truths: What is the evidence on the risks associated with smoking in cars carrying children?. CMAJ.

[41] Ní Shé É., Morton S., Lambert V., Ní Cheallaigh C., Lacey V., Dunn E., Loughnane C., O’Connor J., McCann A., Adshead M. (2019). Clarifying the mechanisms and resources that enable the reciprocal involvement of seldom heard groups in health and social care research: A collaborative rapid realist review process. Health Expect..

[42] Ní Shé É., Keogan F., McAuliffe E., O’Shea D., McCarthy M., McNamara R., Cooney M.T. (2018). Undertaking a Collaborative Rapid Realist Review to Investigate What Works in the Successful Implementation of a Frail Older Person’s Pathway. Int. J. Environ. Res. Public Health.

[43] Teeling S.P., Davies C., Barnard M., O’Connor L., Coffey A., Lambert V., McNamara M., Tuohy D., Frawley T., Redmond C. (2021). A Rapid Realist Review of Quality Care Process Metrics Implementation in Nursing and Midwifery Practice. Int. J. Environ. Res. Public Health.

[44] Archer M.S. (1996). Culture and Agency: The Place of Culture in Social Theory.

[45] Sayer A. (2000). Realism and Social Science.

[46] Pawson R., Greenhalgh T., Harvey G., Walshe K. (2005). Realist review–a new method of systematic review designed for complex policy interventions. J. Health Serv. Res. Policy.

[47] Leech N.L., Onwuegbuzie A.J. (2009). A typology of mixed methods research designs. Qual. Quant..

[48] Schifferdecker K.E., Reed V.A. (2009). Using mixed methods research in medical education: Basic guidelines for researchers. Med. Educ..

[49] Gilmore B., McAuliffe E., Power J., Vallieres F. (2019). Data Analysis and Synthesis Within a Realist Evaluation: Toward More Transparent Methodological Approaches. Int. J. Qual. Methods.

[50] Salter K.L., Kothari A. (2014). Using realist evaluation to open the black box of knowledge translation: A state-of-the-art review. Implement. Sci..

[51] Manzano A. (2016). The Craft of Interviewing in Realist Evaluation. Evaluation.

[52] McCormack B., McGowan B., McGonigle M., Goode D., Black P., Sinclair M. (2014). Exploring ‘self’ as a person-centred academic through critical creativity: A case study of educators in a school of nursing. Int. Pract. Dev. J..

[53] Mannay D. (2010). Making the familiar strange: Can visual research methods render the familiar setting more perceptible?. Qual. Res..

[54] Mannay D. (2016). Visual, Narrative and Creative Research Methods.

[55] Kara H. (2015). Creative Research Methods in the Social Sciences.

[56] Gauntlett D. (2007). Creative Explorations: New Approaches to Identities and Audiences.

[57] Ellingson L.L. (2009). Engaging Crystallization in Qualitative Research.

[58] Pawson R. (2013). The Science of Evaluation: A Realist Manifesto.

[59] Mason J. (2002). Qualitative Researching.

[60] Bahn S., Barratt-Pugh L. (2013). Getting reticent young male participants to talk: Using artifact-mediated interviews to promote discursive interaction. Qual. Soc. Work..

[61] Loeffler T.A. (2005). Looking deeply in: Using photo-elicitation to explore the meanings of outdoor education experiences. J. Exp. Educ..

[62] Stanczak G.C. (2007). Visual Research Methods: Image, Society, and Representation.

[63] Szto P., Furman R., Langer C. (2005). Poetry and photography: An exploration into expressive/creative qualitative research. Qual. Soc. Work..

[64] Foster V. (2007). The art of empathy: Employing the arts in social inquiry with poor, working-class women. Soc. Justice.

[65] Sutton B. (2011). Playful cards, serious talk: A qualitative research technique to elicit women’s embodied experiences. Qual. Res..

[66] Rushmer R.K., Hunter D.J., Steven A. (2014). Using interactive workshops to prompt knowledge exchange: A realist evaluation of a knowledge to action initiative. Public Health.

[67] Phillips L.D., Phillips M.C. (1993). Facilitated work groups: Theory and practice. J. Oper. Res. Soc..

[68] Kaner S. (2007). Facilitator’s Guide to Participatory Decision Making.

[69] Post A., Narayan T. A Design for Manufacturability Workbook 2006. Proceedings of the 2006 ASEE Annual Conference & Exposition.

[70] De Paolo C.A., Wilkinson K. (2014). Using word clouds for analyzing qualitative assessment data. Tech. Trends.

[71] McNaught C., Lam P. (2010). Using Wordle as a Supplementary Research Tool. Qual. Rep..

[72] Coats E., Dewing J., Titchen A. (2006). Opening Doors on Creativity: Resources to Awaken Creative Working.

[73] McCormack B., Dewar B., Wright J., Garbett R., Harvey G., Ballantine K. (2006). A Realist Synthesis of Evidence Relating to Practice Development.

[74] McCormack B., Titchen A. (2006). Critical creativity: Melding, exploding, blending. Educ. Action Res..

[75] Buckley C. (2017). Knowing me, knowing you: Using creative methods to highlight challenges and discover identity and context in an action research study. Int. Pract. Dev. J..

[76] Fine P., Leung A., Francis J., Louca C. (2018). The Use of Picture Cards to Elicit Postgraduate Dental Student Feedback. Dent. J..

[77] Greenhalgh T., Humphrey C., Hughes J., MacFarlane F., Butler C., Pawson R. (2009). How do you modernize a health service? A realist evaluation of whole-scale transformation in London. Milbank Q..

[78] Rycroft-Malone J., Fontenla M., Bick D., Seers K. (2010). A realistic evaluation: The case of protocol-based care. Implement. Sci..

[79] Cardiff S., McCormack B., McCance T. (2018). Person-centred leadership: A relational approach to leadership derived through action research. J. Clin. Nurs..

[80] Reynolds M., Vince R. (2017). Organizing Reflection.

[81] Barton L. (2005). Emancipatory research and disabled people: Some observations and questions. Educ. Rev..

[82] Rolfe G., Freshwater D., Jasper M. (2001). Critical Reflection for Nursing and the Helping Professions: A User’s Guide.

[83] Prior S.J., Mather C., Ford K., Bywaters D., Campbell S. (2020). Person-centred data collection methods to embed the authentic voice of people who experience health challenges. BMJ Open Qual..

[84] Beringer A., Fletcher M. (2011). Developing practice and staff: Enabling improvement in care delivery through participatory action research. J. Child Health Care.

[85] Rainford J. (2020). Confidence and the effectiveness of creative methods in qualitative interviews with adults. Int. J. Soc. Res. Methodol..

[86] Dewing J., McCormack J., Titchen A. (2015). Practice Development Workbook for Nursing, Health and Social Care Teams.

[87] Pearson M., Brand S.L., Quinn C., Shaw J., Maguire M., Michie S., Briscoe S., Lennox C., Stirzaker A., Kirkpatrick T. (2015). Using realist review to inform intervention development: Methodological illustration and conceptual platform for collaborative care in offender mental health. Implement. Sci..

[88] Bunn F., Goodman C., Jones P.R., Russell B., Trivedi D., Sinclair A., Bayer A., Rait G., Rycroft-Malone J., Burton C. (2017). Managing diabetes in people with dementia: A realist review. Health Technol. Assess..

[89] Taylor J., Barker A., Hill H., Haines T.P. (2015). Improving person-centered mobility care in nursing homes: A feasibility study. Geriatr. Nurs..

[90] Tennant E., Miller E., Costantino K., De Souza D., Coupland H., Fotheringham P., Eastwood J. (2020). A critical realist evaluation of an integrated care project for vulnerable families in Sydney, Australia. BMC Health Serv. Res..

[91] Cook N.F., McConnell D., Teeling S.P., Dewing J., McCormack B., McCance T. (2021). Multiple and Mixed Methods Research. Person-centred Nursing Research: Methodology, Methods, and Outcomes.

[92] Manley K., Sanders K., Cardiff S., Webster J. (2011). Effective workplace culture: The attributes, enabling factors and consequences of a new concept. Int. Pract. Dev. J..

[93] Finney L. (2013). Our Shared Purpose: A Practical Guide.

[94] Bolton G. (2014). Reflective Practice Writing and Professional Development.

[95] Williamson G., Bellman L., Webster R. (2012). Action Research in Nursing and Healthcare.

[96] Waterman H., Tillen D., Dickson R., De Koning K. (2001). Action Research a systematic review and guidance for assessment. Health Technol. Assess..

[97] Selvam S.G., Collicutt J. (2013). The ubiquity of the character strengths in African traditional religion: A thematic analysis. Well-Being and Cultures.

[98] Hammersley M., Atkinson P. (2007). Ethnography: Principles in Practice.

[99] Denning J., Verschelden C. (1993). Using the focus group in assessing training needs: Empowering child welfare workers. Child Welf..

[100] Kitzinger J. (1995). Introducing focus groups. Br. Med. J..

[101] Blamey A., Mackenzie M. (2007). Theories of change and realistic evaluation: Peas in a pod or apples and oranges?. Evaluation.

